# New Adenovirus in Bats, Germany

**DOI:** 10.3201/eid1512.090646

**Published:** 2009-12

**Authors:** Michael Sonntag, Kristin Mühldorfer, Stephanie Speck, Gudrun Wibbelt, Andreas Kurth

**Affiliations:** Robert Koch Institute, Berlin, Germany (M. Sonntag, A. Kurth); Leibniz Institute for Zoo and Wildlife Research, Berlin (K. Mühldorfer, S. Speck. G. Wibbelt)

**Keywords:** Virus isolation, vespertilionid bat, adenovirus, VIDISCA PCR, Pipistrellus pipistrellus, generic PCR, Mastadenovirus, virus transmission, Chiroptera, viruses, dispatch

## Abstract

We tested 55 deceased vespertilionid bats of 12 species from southern Germany for virus infections. A new adenovirus was isolated from tissue samples of 2 *Pipistrellus pipistrellus* bats, which represents the only chiropteran virus isolate found in Europe besides lyssavirus (rabies virus). Evidence was found for adenovirus transmission between bats.

Since the recent discoveries of Ebola virus, Henipavirus, and severe acute respiratory syndrome–associated coronavirus infections, interest in the role of bats as hosts for pathogens has markedly increased (*1*). With the exception of worldwide studies on bat lyssaviruses (*2*), most virologic investigations in bats have been limited to a particular zoonotic agent implicated in a geographically localized disease outbreak (*3*–*5*). In the remaining studies, various medically less important viruses have been discovered in bats in the Americas, Africa, Asia, and Australia (*1*,*6*). As a result of increasing research efforts regarding bats and infectious diseases in Europe, 2 new virus groups were recently detected, namely beta- and gammaherpesviruses in organ tissue (*7*) and group I coronaviruses in feces of European vespertilionid bats (*8*). However, because all bat species in Europe/Germany are protected by strict regulations, the acquisition of suitable samples for virus isolation is rather challenging in comparison to most other parts of the world.

## The Study

We performed an extensive search for unknown viruses in 55 German vespertilionid bats based on both generic PCR assays and virus isolation techniques, as part of a broader study investigating histopathologic changes in German bats in association with infectious pathogens. Dead or moribund bats of 12 species (*Barbastella barbastellus, Eptesicus nilssoni, E. serotinus, Myotis daubentonii, M. mystacinus, Nyctalus leisleri, N. noctula, Pipistrellus kuhli, P. nathusii, P. pipistrellus, Plecotus auritus*, and *Vespertilio murinus*) were collected at certified bat rehabilitation centers in southern Germany and were investigated macroscopically, bacteriologically, and histologically.

For virologic examination, homogenized organ tissue was inoculated onto VeroE6/7 cells and monitored daily for cytopathic effects. Remaining tissue material was used for RNA/DNA extraction and further molecular analysis by generic PCR assays to detect members of several virus families including flaviviruses, hantaviruses, coronaviruses, orthomyxoviruses, and paramyxoviruses. The species of bat involved was determined by amplification and sequencing of the cytochrome B (*cytB*) gene, a standard technique for species identification (*9*).

**Figure 1:**
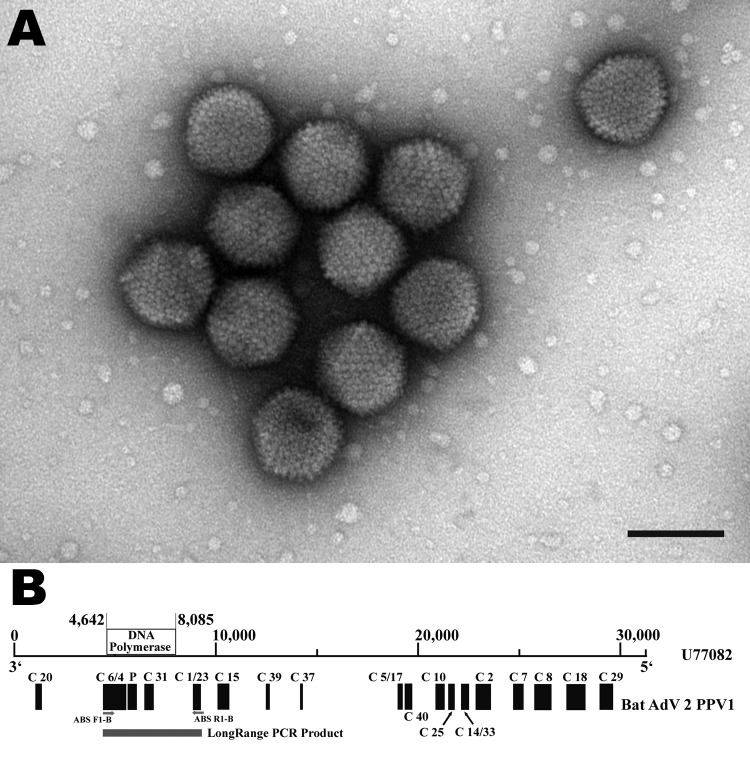
A) Electron micrograph of adenovirus particles isolated from *Pipistrellus pipistrellus* bat 199/07, Germany. Negatively stained with 1% uranyl acetate. Virus particles were 70–90 nm in diameter with an icosahedral shape. Scale bar = 100 nm. B) Schematic representation of the genomic fragments obtained from bat adenovirus 2 (GenBank accession no. FJ983127) in correspondence to canine adenovirus 2 strain Toronto A26/61 (GenBank accession no. U77082). Genomic fragments were generated by generic adenovirus-specific PCR (*10*) and a virus discovery based on cDNA–amplified fragment length polymorphism PCR method (*11*). Partial sequence of the DNA polymerase gene was generated from LongRange PCR product. Purified PCR products were directly sequenced by using the BigDye Terminator Cycle Sequencing ready Reaction kit (Applied Biosystems, Foster City, CA, USA) and analyzed on an ABI 3770 automatic sequencer (Applied Biosytems). C, clone; P, ≈550-bp nested PCR product.

Of the tested samples from 55 bats, virus was initially detected in only 2 adult common pipistrelles (*P. pipistrellus*, nos. 198/07 and 199/07). A cytopathic effect was detected in Vero E6/7 cells after the second passage, indicating the presence of virus in the cell culture. Purified supernatant of these cell cultures was subjected to negative-staining electron microscopy, which showed numerous adenovirus-like particles (Figure 1, panel A). The family *Adenoviridae* was verified by the first reaction of a generic adenovirus-specific nested PCR (*10*). The obtained sequence of a fragment of the DNA polymerase gene (≈550 bp) indicated that the viruses were a novel virus type within the genus *Mastadenovirus* and was tentatively named bat adenovirus 2 (bat AdV-2) strain *P. pipistrellus* virus 1 (PPV1).

**Figure 2:**
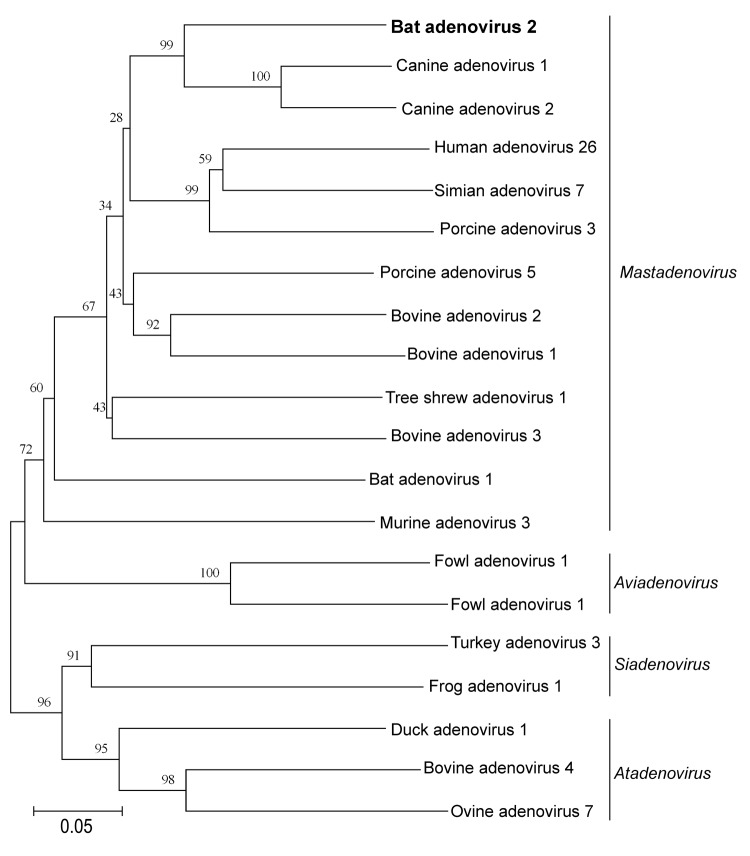
Phylogenetic tree constructed by using a multiple alignment of ≈550-bp amplicons, consisting of the partial DNA polymerase gene of the novel bat adenovirus 2 strain *Pipistrellus pipistrellus* virus 1 (in **boldface**; GenBank accession no. FJ983127) and selected members of the family *Adenoviridae*, Germany. Alignment was analyzed with the neighbor-joining method and p-distance model in MEGA4 (www.megasoftware.net). Bootstrap values (1,000 replicates) >35% are indicated at the branch nodes. Branch length is proportional to evolutionary distance (scale bar). Adenovirus genera are indicated.

To obtain additional sequence information of bat AdV-2, a random PCR method (virus discovery based on cDNA–amplified fragment length polymorphism) (*11*) was applied, which showed >20 adenovirus sequences distributed over the genome (Figure 1, panel B). The partial sequence of the bat AdV-2 DNA polymerase (3,408 bp; GenBank accession no. FJ983127) was obtained after LongRange PCR by using the Expand Long Range dNTPack (Roche, Mannheim, Germany) according to the manufacturer’s directions and the following 2 primers: ABS F1-B (5′-AAAAgAggCAAAgCAAgACAgTgg-3′) and ABS R2-B (5′-ggCgggCAACAAAgACCTCA-3′). After repeated sequence analysis of the partial DNA polymerase gene for validation, we found that the identities of bat AdV-2 PPV1 to closely related adenoviruses ranged from 68% to 74% on nucleic acid level (Table 1), with the closest relationship found to canine adenoviruses 1 and 2. So far, the only other adenovirus in bats has been accidentally isolated from primary bat kidney cells of a healthy Ryukyu flying fox (*Pteropus dasymallus yayeyamae*) (*12*), which proved to have a rather distant phylogenetic relationship to bat AdV-2 (Figure 2).

**Table 1:** Sequence identities (%) of partial DNA polymerase gene (3,408 bp) between bat AdV-2 and selected adenoviruses, Germany*

Virus	Canine AdV-2	Canine AdV-1	Porcine AdV-5	Human AdV-26	Simian AdV-7	Bovine AdV-2	Tree shrew AdV-1
Bat AdV-2 PPV1	74	72	70	69	69	68	68

*AdV, adenovirus; PPV1, *Pipistrellus pipistrellus* virus 1.

On the basis of newly acquired sequence information, we designed a specific real-time TaqMan PCR to detect bat AdV-2 (ABS forward 5′-CACAAgTggTgTCTTTgAgAgCA-3′, ABS reverse 5′-AgAgggATACAAACTgATggAAACA-3′, ABS TM 6FAM-CTAACTTggCTggTggAgTgCgAAAC-q). Cycler conditions were as follows: predenaturation (95°C for 10 min), 45 amplification cycles (95°C for 30 s, 61°C for 30 s, 72°C for 30 s), and final extension (72°C for 10 min).

After screening all 55 bats from Germany of 12 species, comprising an additional 11 common pipistrelles, an identical adenovirus was detected in 1 additional common pipistrelle. Moreover, the tissue tropism of bat AdV-2 was investigated in all 3 infected bats (Table 2). Of all tested organs, bat AdV-2 was detected in high DNA copy numbers in the intestine of all 3 bats with lesser DNA copy numbers in liver and kidneys, whereas the other organs contained little or no adenovirus DNA. Unfortunately, due to advanced tissue decomposition in most of the organs, including liver, kidneys, and intestines, thorough histopathologic examination of the 3 bats was markedly impaired.

**Table 2:** Distribution of DNA copy numbers of bat AdV-2 in different organs of similar size of infected *Pipistrellus pipistrellus* bats, Germany*†

Bat no.	Intestine	Liver	Kidney	Lung	Heart	Brain	Spleen
198/07	+++++	+++	++++	+	–	–	ND
199/07	++++++	++++	+++	+	+	–	ND
200/07	+++++++	+++	++	+	–	–	++

*AdV, adenovirus; +, positive; –, negative; ND, not determined. †Analyzed by bat AdV-2 PPV1–specific TaqMan PCR. Increasing virus DNA copy numbers are indicated by the number of plus signs, which are equivalent to multiples of 3 cycle threshold values, starting with values from 37.0 to 40.0.

## Conclusions

In contrast to Maeda et al. (*12*), who postulated the necessity of primary bat cells to isolate DNA virus from chiroptera, our isolation of a DNA virus from an European bat in a permanent cell line (monkey kidney cells) proved the opposite. We believe that the rare detection and isolation of viruses might be attributed to the fast natural degradation of bats of the suborder Microchiroptera in comparison to that of other animal carcasses, most likely due to their extremely low weight (2–10 g). Although viruses were not detected by various generic PCR assays from homogenized frozen tissue samples, we isolated a novel virus from a hibernating insectivorous bat species. This virus was detected in high DNA copy numbers in the intestine of 3 bats that died of natural causes.

The fact that no other viral or bacterial agents were detected in these animals suggests a clinical correlation to the isolated adenovirus. Moreover, all 3 bats belonged to the same species and were of similar age. Several days before their death, they were found moribund and subsequently admitted together to the rehabilitation center, which highlights the strong likelihood of infection in the colony of origin. Cross-contamination during tissue preparation can be excluded because sterilized instruments were used for each animal, and after every incision, instruments were cleaned with 70% ethanol and a Bunsen burner flame to destroy adhering tissue remnants.

Various adenovirus types of the genus *Mastdenovirus* infect a range of different mammals and cause respiratory, ocular, and gastrointestinal diseases. Here, in all 3 infected bats, the highest copy number of adenovirus DNA was detected in the intestine, which suggests a correlation with a gastrointestinal disease. The host range of mastadenoviruses is known to be limited to a single (or a few closely related) mammalian species (*13*) with a probable co-evolution between virus and their hosts (*14*). The acquired partial sequence of the bat AdV-2 DNA polymerase with the closest relation to canine adenovirus (only 74% at the nucleic acid level) and the isolation from a new animal host suggests that this virus is a new adenovirus species within the genus *Mastadenovirus*. A comparison to the only other adenovirus found in a bat (flying fox, order Megachiroptera) with the available sequence information of a ≈550-bp fragment of the DNA polymerase gene showed their distant relationship. This strict separation reflects either the co-evolutionary development between the 2 adenoviruses (bat AdV-1 FBV1, bat AdV-2 PPV1) and their host families Pteropodidae and Vespertilionidae or a host switch of the virus originating from a yet-undetermined vertebrate host. To elucidate this problem, further research will be necessary.

In conclusion, we isolated a new virus from free-ranging vespertilionid bats, which represents the only chiropteran virus isolate besides lyssavirus (rabies) found in Europe. Moreover, the detection of this chiropteran virus can be connected with its transmission between individual bats living in close proximity to other bats.
